# Usability Issues of Clinical and Research Applications of Virtual Reality in Older People: A Systematic Review

**DOI:** 10.3389/fnhum.2020.00093

**Published:** 2020-04-08

**Authors:** Cosimo Tuena, Elisa Pedroli, Pietro Davide Trimarchi, Alessia Gallucci, Mattia Chiappini, Karine Goulene, Andrea Gaggioli, Giuseppe Riva, Fabrizia Lattanzio, Fabrizio Giunco, Marco Stramba-Badiale

**Affiliations:** ^1^Applied Technology for Neuro-Psychology, IRCCS Istituto Auxologico Italiano, Milan, Italy; ^2^Department of Psychology, Catholic University of the Sacred Hearth, Milan, Italy; ^3^Faculty of Psychology, University of eCampus, Novedrate, Italy; ^4^IRCCS Fondazione Don Carlo Gnocchi, Milan, Italy; ^5^Department of Geriatrics and Cardiovascular Medicine, IRCCS Istituto Auxologico Italiano, Milan, Italy; ^6^Scientific Direction, IRCCS INRCA, Ancona, Italy

**Keywords:** aging, assessment, rehabilitation, usability, user-experience, virtual reality

## Abstract

Aging is a condition that may be characterized by a decline in physical, sensory, and mental capacities, while increased morbidity and multimorbidity may be associated with disability. A wide range of clinical conditions (e.g., frailty, mild cognitive impairment, metabolic syndrome) and age-related diseases (e.g., Alzheimer's and Parkinson's disease, cancer, sarcopenia, cardiovascular and respiratory diseases) affect older people. Virtual reality (VR) is a novel and promising tool for assessment and rehabilitation in older people. Usability is a crucial factor that must be considered when designing virtual systems for medicine. We conducted a systematic review with Preferred Reporting Items for Systematic reviews and Meta-analysis (PRISMA) guidelines concerning the usability of VR clinical systems in aging and provided suggestions to structure usability piloting. Findings show that different populations of older people have been recruited to mainly assess usability of non-immersive VR, with particular attention paid to motor/physical rehabilitation. Mixed approach (qualitative and quantitative tools together) is the preferred methodology; technology acceptance models are the most applied theoretical frameworks, however senior adapted models are the best within this context. Despite minor interaction issues and bugs, virtual systems are rated as usable and feasible. We encourage usability and user experience pilot studies to ameliorate interaction and improve acceptance and use of VR clinical applications in older people with the aid of suggestions (VR-USOP) provided by our analysis.

## Introduction

Life expectancy is rapidly increasing and is expected to rise in the years to come, thereby creating an aging population. However, a significant proportion of older people may develop frailty, multi-morbidity, and disability causing a significant impact both on their quality of life and also on health care and social costs (Lutz et al., 2008; World Health Organization, 2015). Aging is associated with physiological changes (e.g., apoptosis, senescence, inflammation) that may lead to systemic alterations (Flatt, 2012). This potential decline may involve sensory, mental, and physical functioning thus leading to-increased morbidity, multi-morbidity, disability, and mortality (World Health Organization, 2015). On the other hand, motor skills, visual, hearing, proprioception, and cognitive abilities (e.g., memory) may be reduced even in healthy older people (Kuehn et al., 2017). In addition, aging hampers psychosocial well-being by adding new developmental tasks or situations (e.g., isolation; Steptoe et al., 2015). In particular, the prevalence of Alzheimer's disease, cancer, chronic obstructive pulmonary disease, maculopathy, osteoarthritis, osteopenia, Parkinson's disease, periodontitis, rheumatoid arthritis, sarcopenia, cardiovascular diseases, and type 2 diabetes increases with age (Tolosa et al., 2006; Dubois et al., 2010; Marengoni et al., 2011; Edwards et al., 2015; Steenman and Lande, 2017; Yakaryilmaz and Öztürk, 2017; Franceschi et al., 2018). Additionally, several clinical conditions may jeopardize the well-being of older people, such as mild cognitive impairment, frailty, or metabolic syndrome (Fried et al., 2001; Petersen, 2004; Portet et al., 2006; Huang, 2009; Xue, 2011; Fedarko, 2012). The main priority of successful management of aging is enabling older people to be healthy, active, and autonomous for as long as possible (World Health Organization, 2002). Accordingly, functional decline is one of the key issues to be managed (World Health Organization, 2015). Among other practices, the use of assistive health technology (AHT; i.e., technologies devoted to maintain or improve functionality, autonomy and well-being) or medical devices (MD; i.e., technologies used for prevention, diagnosis and treatment) may also produce a beneficial effect in older people (Garçon et al., 2016); however, a critical aspect is to ensure accessibility and use of these technologies in the older population (World Health Organization, 2015; Beard et al., 2016).

Virtual reality (VR) is one of the emerging AHT and MD in the field of aging, frailty, and disability (Lange et al., 2010; Bohil et al., 2011). VR is defined as a system based on an interactive computer-simulated 3D environment (Gorini and Riva, 2008), which incorporates mainly auditory and visual feedback, and sometimes also haptic. VR can be divided in non-immersive, semi-immersive, and fully immersive systems (Mujber et al., 2004). The non-immersive system is a desktop-based VR with low interaction (e.g., keyboard, joypad) and immersion (e.g., PC, tablet). The semi-immersive system consists of a large monitor/projector with moderate immersion and interaction (e.g., Kinect, data gloves). The immersive system is characterized by the use of tools such as a head-mounted display (HMD) or the cave automatic virtual environment (CAVE) that enables a high degree of interaction (e.g., trackers) and immersion in the virtual environment (VE). Additionally, VR can be conceptualized as a continuum between reality and virtuality, where some aspects of VE are mixed with the real environment (augmented reality) or vice-versa (augmented virtuality) (Milgram et al., 1995). The sensorimotor channels connected to the VR define the degree of immersion; the psychological consequence of immersion on perception is the sense of presence that felt through being in the VE or, alternatively, the “*perceptual illusion of non-mediation*” with the VE (Riva, 2008; Bohil et al., 2011). Moreover, mobile applications (e.g., tablet) with tracking systems of the user and/or visors (e.g., Google Cardboard) can be considered mobile VR that allow for different degrees of immersion and interaction with the VE (Pallavicini et al., 2015; Fang et al., 2017).

VR has several requirements for motor and cognitive neurorehabilitation interventions: repetitive practice, feedback about performance, multimodal stimulation, and controlled, secure, and ecologically valid environments (Bohil et al., 2011). It is possible to control and manipulate tailored exercises within meaningful and motivating environments using virtual environments, i.e., transformation of flow (Riva et al., 2006). For these reasons, VR has been utilized for rehabilitation in different fields and, particularly, after stroke. Accordingly, guidelines have recently included the use of VR for both motor and cognitive rehabilitation in patients who suffered a stroke (ISO, 2016b; Winstein et al., 2016). However, access to this kind of technology may be limited by the lack of accessibility in the older population, as compared to other AHT and MD (World Health Organization, 2015). For instance, VR in the context of stroke rehabilitation is facing challenges concerning end-users' interaction, such as feasibility of VR training, lack of functional relevance, patient frustration to feedback, and lack of integration of environmental factors that link to motor performance (Teo et al., 2016).

On the macroscopic level, access to AHT and MD is limited by socio-demographic and economic terms, while on the microscopic level, access is the use itself of a device. Indeed, according to the MOLD-US framework (Wildenbos et al., 2018), the use of technology among older people is hampered by different barriers: (1) cognitive (e.g., reduced working memory, spatial cognition, attention, language, and reasoning) and motivational (e.g., self-efficacy, self-confidence, benefits identification, computer literacy, integration in daily life) that affect the use with errors, efficiency, learnability, memorability and satisfaction; (2) physical (e.g., motor speed, flexibility, hand-eye coordination, strength) and perception (e.g., vision, auditory, haptic) that influence errors and efficiency. According to Nielsen (Nielsen, 2012), usability is defined by learnability (is it easy to accomplish the task?), efficiency (once learned, is the user fast in performing the task?), memorability (is the user able to reestablish proficiency with the design after a period of stop?), errors (how many errors does the user make?) and satisfaction (how pleasant is the design?). Along with usability (i.e., easiness and pleasure), the technology should provide the attributes needed by the user (i.e., utility). Usability can be assessed by a means of a wide range of methods, such as the system usability scale (SUS), heuristic evaluation, cognitive and pluralistic walkthrough, formal usability, pluralistic, consistency, and standard inspections (Brooke, 1986; Nielsen, 1994).

Nevertheless, usability tends to focus more on the task rather than on the experience (Vermeeren et al., 2010). Indeed, researchers investigating user experience (UX) point out a role of factors that go beyond the technology and its usability/usefulness. UX facets embrace emotion and affective reactions toward the technology and experiential, hedonic, holistic, and aesthetic factors. The interaction with a technology is “*a subjective, situated, complex, and dynamic encounter*” (Hassenzahl and Tractinsky, 2006). If it is true that satisfaction plays a critical role in usability, UX takes into account emotions, motivation, and expectation of human-computer interaction (Vermeeren et al., 2010). For instance, the user experience questionnaire (UEQ) aims at evaluating six factors: attractiveness, perspicuity, efficiency, dependability, stimulation, and novelty (Laugwitz et al., 2008), or the usability metric for user experience (UMUX) taps UX facets of usability (Finstad, 2010). Additionally, 96 UX methods (http://www.allaboutux.org/all-methods) have been identified in the UX research field (Vermeeren et al., 2010). UX methods range from qualitative to quantitative techniques, target technology, period of assessment (e.g., developmental, conceptual), time, information source (e.g., experts, specific users, individual, group), and location (e.g., lab, online, field). Methods range from semantic differential, checklists, heuristics, think-aloud, psychophysiological measures, self-report, questionnaires, *in situ* observation, and video analysis (Vermeeren et al., 2010). A critical aspect of UX is the prototype development (Novak, 2008), which follows the concept (idea) and pre-production (demo) phases and precedes production & localization (development), Alpha, Beta, and Gold/post-production phases.

A wide range of theories have been proposed to understand and explain user acceptance and use of technology (for a literature review see Taherdoost, 2018). The most inclusive model is the unified theory of acceptance and use of technology model (UTAUTM) (Venkatesh et al., 2003), which includes the technology acceptance model (TAM), theory of reasoned action, theory of planned behavior (TPB), combined TAM and TPB, model of PC utilization, the diffusion of innovation model, motivational model, and social cognitive theory. In this model, the significant factors are: effort, expectancy, performance expectancy, social influence, and facilitating conditions. Interestingly, starting from the TPB (Fishbein and Ajzen, 1975), TAM (Davis et al., 1989), and UTAUTM (Chen and Shou, 2014), developed the senior technology acceptance model (STAM). Controlling age, gender, educational level, and economic status, their model included gerontotechnology self-efficacy and anxiety, facilitating health conditions, cognitive abilities, social relationships, attitude to life and satisfaction, and physical functioning as factors that influenced perceived usefulness, usage behavior, and perceived ease of use, which in turn affects general attitude toward the use. A similar model (senior citizens' acceptance of information systems; SCAIS) was developed by Phang et al. (2006). This model takes into account preference for human contact, self-actualization, resource saving, anxiety, computer support, physiological decline which influences perceived usefulness, ease of use, internet safe perception and in turn, intention. Another theoretical framework used to approach technology use and acceptance is the user-centered design (UCD). UCD enables technology systems to be made more usable and interactive to end-users, but it can also be applied to assess needs, wants, and limitations of general products (Sebe, 2010; ISO, 2016a; Brox et al., 2017). UCD can be investigated using a variety of qualitative and quantitative methods such as field studies, user requirements analyses, iterative design, usability evaluation, task analyses, focus groups, user interviews, participatory design, and prototypes (Vredenburg et al., 2002). UX can be explored with the playability model (i.e., immersion, socialization, emotion, satisfaction, effectiveness) that is crucial when building games for clinical purposes (Sánchez et al., 2012; Valladares-Rodriguez et al., 2019); emotive design for VR should be followed for designing human-computer interaction systems (see Vredenburg et al., 2002).

Lastly, human-computer interfaces are also conceptualized in terms of architecture and layers needed to provide a service (Tsai et al., 2012; Nikitina et al., 2018). For instance, the user remote console (URC) is a framework used for telemedicine systems to define abstract user interface layers, hubs, and devices. If a researcher wishes to consider a VR AHT or MD for healthcare purposes, in addition to the usability and UX aspects, they may want to assess the sense of presence in the VE. According to the Inner Presence theory (Lee, 2004; Riva and Waterworth, 2014), presence is not necessarily related to media characteristics (e.g., graphic realism) but rather to an everyday life flow that controls actions through a constant intentions-perceptions comparison. In this sense, a VR user may experience the system as usable, as they are able to enact actions thanks to an easy-to-learn interface that tracks user's movements, an understandable game/training structure, and engaging storytelling (Triberti and Riva, 2016). These elements are particularly relevant for videogames and serious games used also for therapeutic purposes (Sáenz-de-Urturi et al., 2015). This conceptualization of presence has relevant consequences when taking clinical practice and change into consideration. VR clinical applications should exploit the transformation of flow (transformative and optimal experience allowed by the sense of presence) to discover and use new and unexpected resources to foster clinical change (Riva et al., 2006, 2016) and consider sensorimotor and cognitive impairments in the old population to customize VR for cognitive (Tuena et al., 2019) or physical (Pedroli et al., 2018) rehabilitation.

This paper aims at systematically reviewing the studies that evaluated feasibility, usability, and UX of assessment and treatment VR systems in healthy aging and age-related clinical conditions. In order to provide an overview of the current research status we analyzed characteristics of participants involved, technological apparatus and use, usability/UX assessments, theoretical framework, and primary outcomes. VR use is classified as the task being accomplished and the training sessions and the aims, which include assessment and rehabilitation. Additionally, we outlined suggestions to assess usability of VR applications for older people in clinical and research contexts.

## Methods

Preferred Reporting Items for Systematic reviews and Meta-Analysis (PRISMA) guidelines were followed (Moher et al., 2009).

### Search Strategy

**Figure 1:**
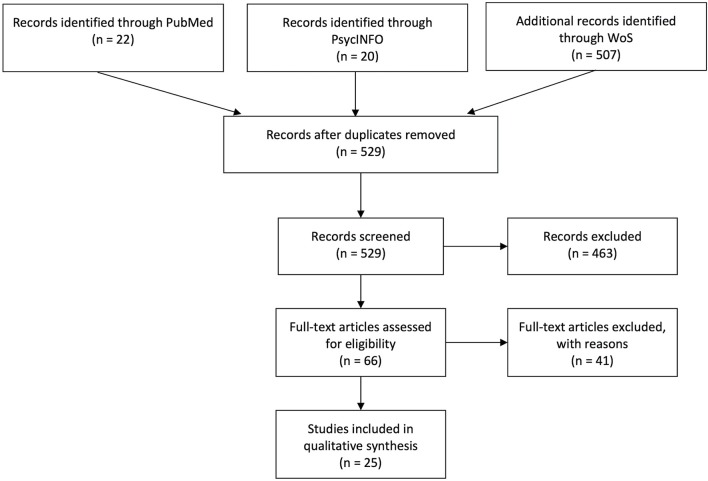
PRISMA flow chart.

Three high-profile databases (PubMed, PsycINFO, and Web of Science) were used to perform the computer-based research on 3 September 2019. The string used to carry out the search (Title/Abstract for PubMed, Topic for Web of Science, Abstract for PsycINFO) was as follows: (“aging” OR “frailty” OR “elder^*^” OR “multimorbidity”) AND (“usability” OR “user experience” OR “UX” OR “user centered design” OR “human centered design” OR “human computer interaction”) AND (“virtual”). The search resulted in 507 articles for Web of Science, 22 for PubMed, and 20 for PsycINFO (total of 529). We made a first selection by reading titles and abstracts after removing duplicates. A total of 66 manuscripts were chosen for full-text screening. This procedure resulted in 25 experimental studies. See the flow diagram (Figure 1) for the paper selection procedure.

### Selection Criteria

Studies concerning the usability, UX, and feasibility of VR (see introduction for definition) systems for assessment/monitoring and rehabilitation/empowerment in healthy and pathological aging were included. In particular, we focused on the age-related clinical conditions in older people. We excluded articles that did not involve usability of VR clinical systems in non-age-related conditions that do not fall into the context of frailty, multimorbidity, or chronicity in aging and with technologies that do not meet VR definition. Additionally, studies for which the full text was not available or for which the abstract lacked basic information for review were removed. Non-English papers, reviews, meeting abstracts, conference proceedings, notes, case reports, letters to the editor, research protocols, patents, editorials, and other editorial materials were also excluded.

### Quality Assessment and Data Abstraction

PRISMA guidelines were strictly followed; search results found by the first author (CT) were shared with the review author (MC) for individual selection of papers in order to reduce the risk of bias, and disagreements were resolved through consensus. The risk of bias for each single study was assessed following the Cochrane guidelines (Higgins et al., 2011) by CT and MC. The research question was formulated according to suggested PICO (Population: older people with age ≥ 65, Intervention: VR for assessment or rehabilitation in age-related conditions and diseases, Comparison: N/A as usability at this time adopt quasi-experimental or pilot study designs (see also risk of bias Supplementary Figure 1), Outcome: measures of usability and acceptance) research question guidelines (Abigail et al., 2014). The Comparison is mainly applied to randomized clinical trials and within our search only one study (Schwenk et al., 2014) satisfied this criterion. Consequently, data extracted from each included study were as follows: reference, year, sample (s), aims, technology, VR training, technology design framework, usability/UX/feasibility assessment tools, primary outcomes, and type (assessment/rehabilitative) of VR system.

## Results

**Figure 2:**
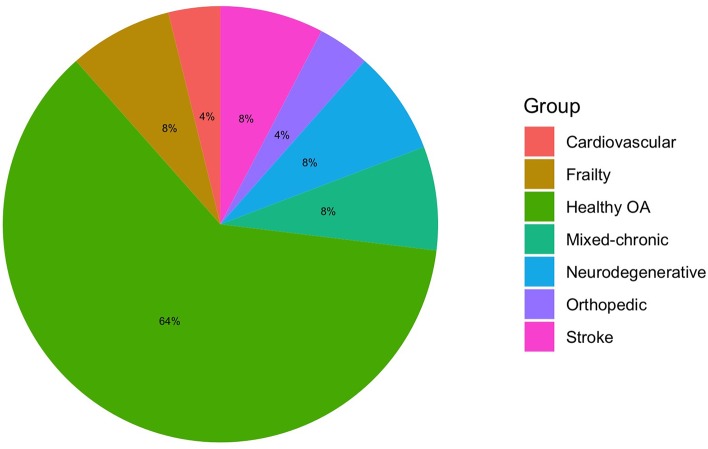
Clinical conditions; OA, older adults.

**Figure 3:**
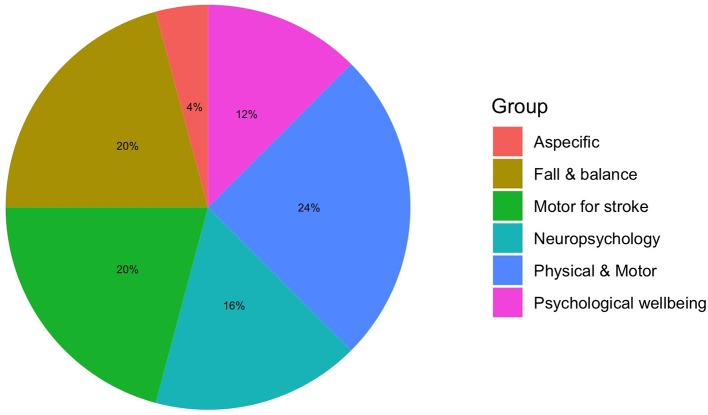
Clinical applications.

**Figure 4:**
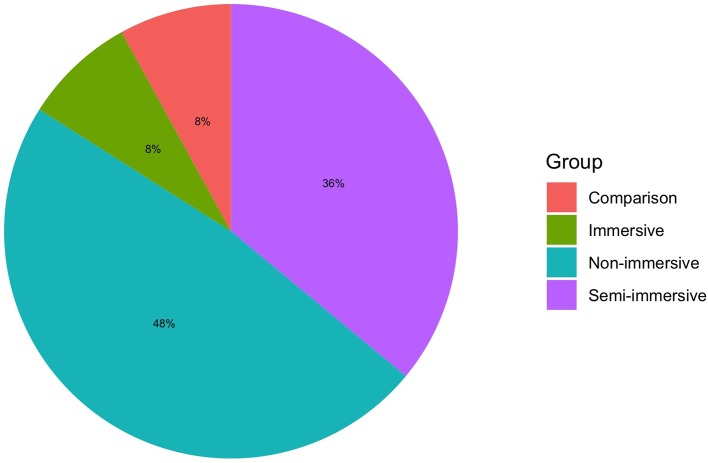
Degree of virtual immersion; VR, virtual reality.

**Figure 5:**
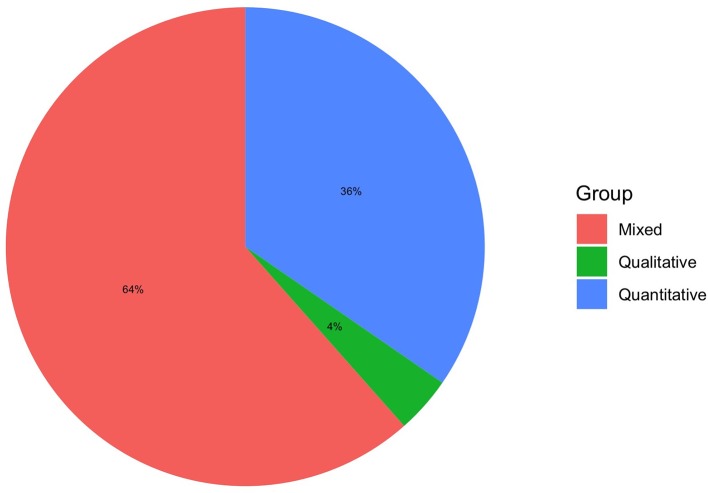
Methodological approach.

**Figure 6:**
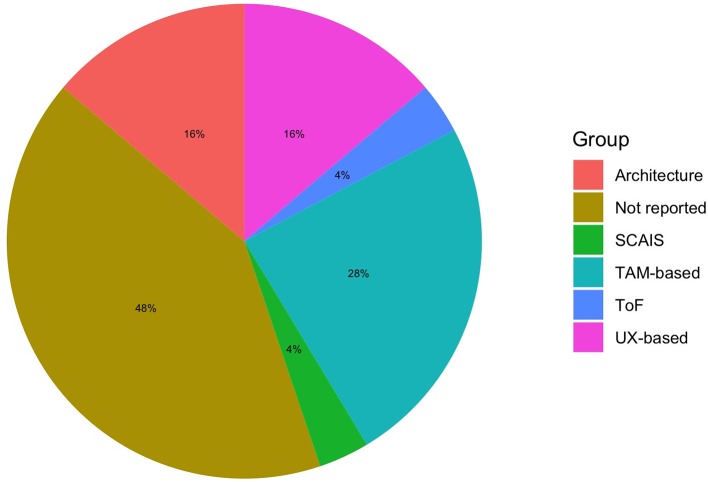
Technology usability and acceptance models; SCAIS, senior citizens acceptance of information systems; TAM, technology acceptance model; ToF, transformation of flow; UX, user experience.

**Figure 7:**
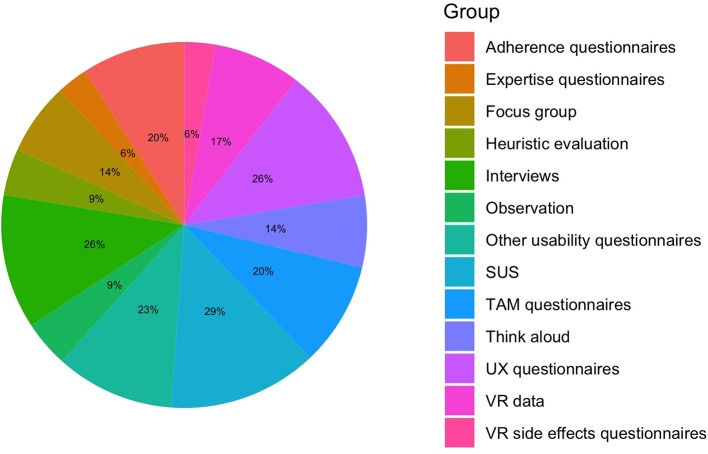
Assessment tools by the total of unique instruments; SUS, system usability scale; TAM, technology acceptance model; UX, user experience; VR, virtual reality.

**Figure 8:**
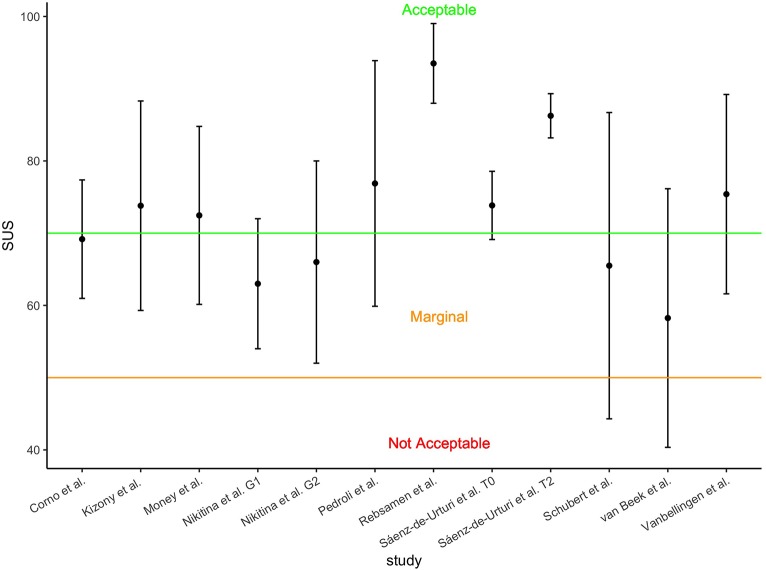
Available mean SUS scores with standard deviations; SUS, system usability scale; G1, group 1; G2 group 2; T0, baseline session; T2, third session.

Our search identified several usability, user experience (UX), and feasibility studies in healthy aging and age-related clinical conditions. A critical aspect of virtual reality (VR) and new technologies is their interaction with humans and in particular, those whose physical, psychological, or social barriers hamper the use of technological devices. The aim of this systematic review was to analyze the current research in the field of usability of clinical VR systems in older people and to provide an overview on this topic. Findings are shown in Table 1 according to reference, year, sample(s), aims of the study, VR technology, VR training, theoretical framework, usability assessment, primary outcomes, and clinical aims. Figures 2–8 summarize the results as well.

**Table 1:** Summary of the studies included.

**References**	**Sample(s)**	**Aims**	**VR technology**	**VR training**	**Design framework**	**Usability assessment tools**	**Primary outcomes**	**Clinical field**
Brox et al. (2017)	10 OA (age range= 66–90, MMSE > 25) with strength/balance impairments and recent illness/surgery	Recording UX and usability of exergame for physical training in OA	Semi-immersive VR with Kinect	Every second week for 3 years to play exergames and participate in the UCD protocol	Senior UCD	UCD-based questionnaire, semi-structured and structured interviews, observation, group discussions	Results show that VR features (e.g., realism, interaction), usability assessment, and physical impairments are critical factors to be taken into account in the older people	R
Valladares-Rodriguez et al. (2019)	64 older people (16 MCI, mean age = 76.87, *SD* = 9.33; 20 AD, mean age = 79.15, *SD* = 4.91; 28 HC, mean age = 75.57, *SD* = 7.14)	Evaluate UX and PX of game-based battery Panoramix	Non-immersive: Samsung Galaxy Note Pro (SM-P900)	Patients played each game twice during two different sessions (45 min)	TAM, playability model and EMOLVE guidelines	Videogame, technology and TAM questionnaires, PSSUQ and PSSUQ-playability-based to administrators	The Panoramix battery is usable and playable by patients, regardless of their socio-cultural level and their technological dexterity	A
Tsai et al. (2012)	52 OA (age range = 64–91)	Exploring the usability of Sharetouch system to encourage social integration for senior users	Semi-immersive VR with infrared LED	One 10 min session	TAM and architecture design	TAM questionnaire	Sharetouch can enrich the users' social network experience through its hardware and software architecture	R
Nikitina et al. (2018)	60 OA (age range = 59–83) with non-to-mild frailty	Exploring the usability of home-based online group training for home physical training (high vs. low social cohesion and interaction vs. individual group)	Non-immersive VR: PC or tablet app (Gymcentral)	8 weeks at least two sessions (30–40 min) per week	SCAIS	SUS, acceptance questionnaire, VR data (e.g., ratio of copresence sessions, time), MOS, PACES	Group exercise app has a high usability and future use. Copresence was found to be related to social cohesion factor	R
Sáenz-de-Urturi et al. (2015)	14 OA (mean age = 81.28, *SD* = 8.94 MMSE = 20–26) with mixed age-related disabilities	Assessing usability of Kinect-based training for physical exercises	Semi-immersive VR with Kinect	Three 9 min sessions	Playability model and architecture design	Heuristic evaluation, videotaping, written observation, think aloud, CEGEQ, modified SUS, physical exercise questionnaire	Results from CEGEQ and SUS suggest a high game playability and usability. End-users and experts are critical during the design phase	R
Pedroli et al. (2018)	5 OA (mean age = 70, SD = 11.70; MMSE > 20)	Evaluating usability, characteristics and experience of the Positive Bike for cognitive and physical therapy in frailty	Immersive VR; CAVE with Cosmed Eurobike 320, Vicon motion tracking system and controller	15 min ride in virtual park with a dual interference task (cognitive vs. physical)	ToF	SUS, flow state scale, semi-structured interview	The Positive Bike was evaluated as usable and provided a positive flow experience	R
Cook and Winkler (2016)	11 OA (mean age = 71.2) who completed the training and 8 OA (mean age = 71.2) non-completers	Exploring usability and engagement of VE for health care	Non-immersive SL environments	Four educational sessions on SL	TAM	TAM-based questionnaire	VE are evaluated as adequate and applicable for health care uses after proper training	R
Castilla et al. (2013)	8 OA (age range = 60–72) with no cognitive deterioration and proper vision and audition level	Development and assessment of Butler, a VR telemedicine system for older people	Non-immersive VR	Conceptual design	Not reported	Group enquiry method, cognitive walkthrough method, and heuristic evaluation method	Older people mental model require accurate user interface design in order to facilitate usability	R
Corno et al. (2014)	10 OA (age ≥ 60. MMSE range: 27–30)	Evaluate the usability of V-MT for executive functions assessment in older people	Immersive VR: HMD with wand	One session with eight tasks of the V-MT	Not reported	Familiarity with technology questionnaire, SSQ, think aloud, SUS, semi-structured usability interview	Usability was found to be crucial for detecting issues of immersive VR (instructions, movements, and realism)	A
Morán et al. (2015)	32 OA (*M* = 64,96; *SD* = 6,31) with no apparent cognitive and functional problems were divided according to their experience of technology	The aim of the study is to discuss usability aspects of Gesture Therapy for stroke rehabilitation according to technology experience	Non-immersive VR with hand sensor	Three games (15 min) in one session	TAM2	TAM-based questionnaire, indirect observation (verbal and non-verbal language)	The study shows that expert and non-expert older people differ in terms of anxiety and enjoyment. Two strategies approach were found for the users (*score and compete* vs. *explore and learn*). Based on these factors, authors provided feedback guidelines for VR trainings	R
Vanbellingen et al. (2017)	13 OA (mean age = 68.2, *SD* = 17.5)	Evaluating the usability, compliance and efficacy of VBT using the LMC to train fine manual dexterity rehabilitation of stroke patients	Non-immersive VR with LMC	Nine training sessions of 30 min, spread out over 3 weeks	Not reported	SUS, VR data (e.g., time), PRPS, interview.	VBT using LMC is a usable rehabilitation tool to train dexterity in stroke patients	R
Trombetta et al. (2017)	10 OA (age range = 61–75)	The aim of the study is to offer a tool (i.e., Motion Rehabe AVE 3D) to improve upper limb motor and balance rehabilitation for stroke patients	Immersive VR with HMD and Kinect and semi-immersive with Smart TV 3D	Motion Rehab AVE 3D contemplates six physical activities	Not reported	Device preference questionnaire and physical training interview	Regarding this pilot study, all participants classified the experience as interesting and excellent for older people. For stroke patients authors suggest semi-immersive apparatus	R
Im et al. (2015)	18 OA (mean age = 64.7, *SD* = 7.27, mean MMSE = 29.06, *SD* = 1)	The aim of the study is to assess a novel 3D ARS balance program	Semi-immersive with Kinect	Ten sessions (30 min, three games) over the course of 4 weeks	Not reported	PRPS, side effects interview (e.g., dizziness, headache, falling and joint pain)	Participants were engaged in the training across the sessions without any adverse effects. 3D ARS is a safe, well-tolerated, motivating and efficacious method	R
Wüest et al. (2014)	16 OA (age > 64, MMSE ≥ 22)	Assessing the usability of a stroke rehabilitation program (REWIRE project) for motor training	Non-immersive VR with force platform	36, 30-min sessions over 12 weeks (five exergames)	Abridged TAM	TAM questionnaire, think aloud, number of drop-outs and completed sessions	The findings revealed high level of acceptance, positive attitude, future use toward the program	R
Rebsamen et al. (2019)	12 OA (mean age = 72.3, *SD* = 4.44, MoCA range = 26–30)	Investigating the feasibility and efficacy of a physical exergame on cardiovascular fitness	Semi-immersive VR: Senso system	4 weeks training with three sessions per week (eight exergames; 30 min circa)	TAM	Think aloud, SUS, TAM questionnaire, enjoyment scale, computer use, VR data	Senso has excellent usability, is fun and well-accepted	R
Plechatá et al. (2019)	36 OA (mean age = 69.47, *SD* = 7.39) vs. 25 YA (mean age = 25.4, *SD* = 5.13)	Assessing age-related differences on immersive vs. non-immersive version of the vSST for episodic memory evaluation	Non-immersive vs. immersive VR: desktop PC and HTC Vive	One session (4–10 min)	Not reported	Ad-*hoc* usability questionnaire	OA memory was worst in the immersive compared to desktop-based VR. YA prefer HMD and generally reported more usability of VR systems. OA did not show a specific preference	A
Money et al. (2019)	15 participants (age range = 50–70)	Exploring and evaluating usability of Falls Sensei 3D for fall prevention	Non-immersive VR	One exergame session (~17 min)	UTAUTM and architecture design	Think aloud, post-experience interview, SUS	Fall Sensei was rated as engaging and feasible serious game for fall prevention	R
Kiselev et al. (2015)	4 participants with fall risk (1 = control group; 3 = intervention group, age > 55)	The aim of the study is to investigate the usability and user acceptance of VR home-based training (i.e., Interactive Trainer) for fall prevention	Semi-immersive VR with Kinect and sensors	6 weeks training (balance exercises)	UCD	Semi-structured interviews, focus group and VR data	Participants stated that the Interactive Trainer was easy to use and exercises challenging but some technical and interaction problems were reported	R
Shubert et al. (2015)	21 OA (mean age = 69.2, *SD* = 5.8) with mixed chronic diseases (no neurodegenerative)	Exploring usability of ST as a possible platform to provide a fall prevention program	Non-immersive VR; VERA software, Kinect and laptop	90 min session of system navigation and physical exercise	Not reported	Debrief survey, think aloud, SUS, interview	OA well-accepted this system and show the potential of ST to provide OEP	R
Schwenk et al. (2014)	33 OA with risk fall. Intervention (mean age = 84.3, SD = 7.3) Control (mean age = 84.9, SD = 6.6; MMSE > 23).	Evaluating the effectiveness and UX of a balance-training program.	Semi-immersive VR with sensors	Training session of 45 min twice a week for 4 weeks	Not reported	GEQ	Training was rated as fun, well-designed and adequate	R
van Beek et al. (2019)	10 PD (mean age = 65.4, *SD* =7.01, Hoehn and Yahr range = 2–4, MoCA range = 22–29)	Evaluating the usability of a dexterity exergame in PD	Non-immersive VR with LMC	Eight 30 min sessions (5 games) for 4 weeks	Not reported	VR data (i.e., time/planned time × 100), PRPS, interview, SUS	Patients showed high adherence, motivation, enjoyment and good usability	R
Desteghe et al. (2017)	15 AF patients (mean age = 69.2, *SD* = 3.7).	The aim of this pilot study was to assess the feasibility and usability of the Health Buddies app in AF patient	Non-immersive (PC, tablet or mobile)	Training lasted every day for 3 months	Not reported	Focus group, UEQ, MMAS-8, MEMS, Helping Hand, VR data	The app was positively rated by its users; nevertheless adherence to medication was low and need user-friendly interface	A
Epelde et al. (2014)	13 medical professionals and 19 orthopedic patients (mean age = 69.31, SD = 7.38)	Assessing the acceptance of a universal remote rehabilitation leaded by avatar	Semi-immersive VR with inertial sensors	One session	URC	Ad *-hoc* usability questionnaire, focus group	Medical professionals were positive regarding the virtual therapists and patients showed good acceptance of the system	R
Fordell et al. (2011)	31 stroke patients (mean age = 74.1, *SD* = 11) with no severe comorbidity and with (*N* = 9) or without (*N* = 22) neglect	Assesses effectiveness and usability of VR-DiSTRO compared to gold-standard neglect assessment	Immersive VR 3D glasses and interaction pen	One sessione (VR 15 min “paper and pencil” 50 min)	Not reported	Ad *-hoc* usability	Patients felt focused, amazed and comfortable with the VR assessment. Any severe side effects were reported.	A
Kizony et al. (2006)	12 OA (mean age = 70.6, *SD* = 4.4) 4 OA patients (3 stroke and 1 spinal stenosis)	Assess usability of TheraGame for neurorehabilitation	Non-immersive VR	One session (30 min) and 2 weeks and half for one stroke patient	Not reported	SUS, SFQ, Borg scale	Both groups showed good level of usability and enjoyment during the session. Also the caregiver who followed the patient during the 2 weeks confirmed the usability.	R

*A, assessment; AD, Alzheimer's disease; AF, atrial fibrillation; ARS, augmented reality system; CAVE, cave automatic virtual environment; CEGEQ, core elements of the gaming experience questionnaire; EMOVLE, emotive virtual learning environment; GEQ, game experience questionnaire; HMD, head-mounted display; HC, healthy controls; LMC, leap motion controller; MOS, medical outcome survey; MEMS, medication event monitoring system; MCI, mild cognitive impairment; MMSE, mini-mental state examination; MoCA, Montreal cognitive assessment; MMAS-8, Morisky medication adherence scale; OA, older adults; OEP, Otago exercise program; PD, Parkinson's disease; PACES, physical activity enjoyment scale; PRPS, Pittsburgh rehabilitation participation scale; PX, player experience; PSSUQ, post-study system usability questionnaire; R, rehabilitation; SL, second life; SCAIS, senior citizens' acceptance of information systems; SFQ, short feedback questionnaire; SSQ, simulator sickness questionnaire; ST, stand tall; SUS, system usability scale; TAM, technology acceptance model; ToF, transformation of flow; UTAUTM, unified theory of acceptance and use of technology model; UX, user experience; UEQ, user experience questionnaire; UCD, user-centered design; URC, user remote console; VBT, videogame-based training; VE, virtual environment; V-MT, virtual multitasking test; VR, virtual reality; vSST, virtual supermarket shopping task; YA, young adults*.

### Which Are the Samples Involved in VR Usability Studies?

The majority of the studies (Kizony et al., 2006; Tsai et al., 2012; Castilla et al., 2013; Corno et al., 2014; Wüest et al., 2014; Im et al., 2015; Morán et al., 2015; Cook and Winkler, 2016; Trombetta et al., 2017; Vanbellingen et al., 2017; Plechatá et al., 2019; Rebsamen et al., 2019) recruited healthy older adults (OA) to assess the usability of clinical VR systems. Two studies collected data from the fifth decade to old age (Kiselev et al., 2015; Money et al., 2019). Nevertheless, in these studies, systems were created for clinical conditions such as stroke (Wüest et al., 2014; Morán et al., 2015; Trombetta et al., 2017; Vanbellingen et al., 2017) or movement disorders (e.g., balance, physical frailty; Pedroli et al., 2018; Money et al., 2019). Indeed, only two studies recruited stroke patients for stroke VR systems (Kizony et al., 2006; Fordell et al., 2011). Sáenz-de-Urturi et al. (2015) and Pedroli et al. (2018) recruited OA and, among these individuals, some had mild or moderate cognitive impairment (O'Bryant et al., 2017). Patients with mixed age-related conditions (e.g., Parkinson's disease, macular degeneration, muscular dystrophy, arthritis, diabetes, hypertension) were recruited in Sáenz-de-Urturi et al. (2015) and Shubert et al. (2015). Frail patients were collected in Nikitina et al. (2018), mixed frail and physical-motor patients in Brox et al. (2017), participants at risk of falling in Schwenk et al. (2014) and Kiselev et al. (2015), mild cognitive impairment (MCI) and Alzheimer's disease (AD) individuals in Valladares-Rodriguez et al. (2019), Parkinson's disease (PD) individuals in van Beek et al. (2019), OA with atrial fibrillation (AF) in Desteghe et al. (2017), and with orthopedics impairments in Epelde et al. (2014). Experts and medical professionals were included in some pilot studies for their opinion on the design or on the VR system (Castilla et al., 2013; Epelde et al., 2014; Morán et al., 2015; Sáenz-de-Urturi et al., 2015; Brox et al., 2017; Desteghe et al., 2017; Valladares-Rodriguez et al., 2019).

### Which are the Aims and the Clinical Fields of the Studies?

All of the studies—except one that was principally devoted to the clinical efficacy of the training (Schwenk et al., 2014)—were mainly designed for the usability, UX and feasibility of VR systems in aging (Tsai et al., 2012; Castilla et al., 2013; Corno et al., 2014; Epelde et al., 2014; Wüest et al., 2014; Im et al., 2015; Kiselev et al., 2015; Morán et al., 2015; Sáenz-de-Urturi et al., 2015; Shubert et al., 2015; Cook and Winkler, 2016; Brox et al., 2017; Desteghe et al., 2017; Trombetta et al., 2017; Vanbellingen et al., 2017; Nikitina et al., 2018; Pedroli et al., 2018; Money et al., 2019; Plechatá et al., 2019; Rebsamen et al., 2019; Valladares-Rodriguez et al., 2019; van Beek et al., 2019). Most of the studies concerned the assessment of therapeutic (i.e., rehabilitative or psychological empowerment) VR systems Tsai et al., 2012; Castilla et al., 2013; Corno et al., 2014; Epelde et al., 2014; Wüest et al., 2014; Im et al., 2015; Kiselev et al., 2015; Morán et al., 2015; Sáenz-de-Urturi et al., 2015; Shubert et al., 2015; Cook and Winkler, 2016; Brox et al., 2017; Desteghe et al., 2017; Trombetta et al., 2017; Vanbellingen et al., 2017; Nikitina et al., 2018; Pedroli et al., 2018; Money et al., 2019; Plechatá et al., 2019; Rebsamen et al., 2019; Valladares-Rodriguez et al., 2019; van Beek et al., 2019, whereas only a few were on assessment or monitoring tools (Fordell et al., 2011; Corno et al., 2014; Desteghe et al., 2017; Plechatá et al., 2019; Valladares-Rodriguez et al., 2019). The intervention/assessment of the studies included were physical-motor (e.g., limb physiotherapy, physical activity, hand motricity; Kizony et al., 2006; Epelde et al., 2014; Schwenk et al., 2014; Wüest et al., 2014; Im et al., 2015; Kiselev et al., 2015; Morán et al., 2015; Sáenz-de-Urturi et al., 2015; Shubert et al., 2015; Brox et al., 2017; Trombetta et al., 2017; Vanbellingen et al., 2017; Nikitina et al., 2018; Pedroli et al., 2018; Money et al., 2019; van Beek et al., 2019), neuro/psychological (Fordell et al., 2011; Tsai et al., 2012; Castilla et al., 2013; Corno et al., 2014; Desteghe et al., 2017; Plechatá et al., 2019; Valladares-Rodriguez et al., 2019), cardiovascular fitness (Rebsamen et al., 2019), or non-specific healthcare applications (Cook and Winkler, 2016).

### Which Are the VR Technologies Used and the Training?

Non-immersive VR (i.e., desktop-based VR, tablet, and mobile app) were used in most of the studies (Kizony et al., 2006; Castilla et al., 2013; Wüest et al., 2014; Morán et al., 2015; Shubert et al., 2015; Cook and Winkler, 2016; Desteghe et al., 2017; Vanbellingen et al., 2017; Nikitina et al., 2018; Money et al., 2019; Valladares-Rodriguez et al., 2019; van Beek et al., 2019). Application exclusively for tablets were used in Valladares-Rodriguez et al. (2019) and multi-device (i.e., PC, tablet or mobile) apps in Castilla et al. (2013), Desteghe et al. (2017), and Nikitina et al. (2018). Semi-immersive VR (i.e., large TV or projector screens with sensors for interaction) systems were tested in some studies (Tsai et al., 2012; Epelde et al., 2014; Schwenk et al., 2014; Im et al., 2015; Kiselev et al., 2015; Sáenz-de-Urturi et al., 2015; Brox et al., 2017; Rebsamen et al., 2019), whereas full immersive VR (i.e., visors or CAVE with interaction devices) was tested only in Fordell et al. (2011), Corno et al. (2014), and Pedroli et al. (2018). Interestingly, Trombetta et al. (2017) compared a semi-immersive vs. an immersive version of the training to evaluate their usability, whereas Plechatá et al. (2019) tested a VR memory test with non-immersive vs. immersive VR.

Sessions lasted from nine to 90 min (mean time = 30 min ca.), ranging from one to 36 sessions spread over the course of 1 single day to 4 years; indeed, usability along with effectiveness of training was tested for three (Vanbellingen et al., 2017), four (Im et al., 2015; Rebsamen et al., 2019; van Beek et al., 2019), six (Kiselev et al., 2015), eight (Nikitina et al., 2018), and 12 weeks (Wüest et al., 2014; Desteghe et al., 2017), and three (Brox et al., 2017) and 4 years (Schwenk et al., 2014). Exergames (i.e., serious games used for balance and fall risk training) were used in most of the motor training (Kizony et al., 2006; Schwenk et al., 2014; Wüest et al., 2014; Kiselev et al., 2015; Sáenz-de-Urturi et al., 2015; Shubert et al., 2015; Brox et al., 2017; Trombetta et al., 2017; Money et al., 2019; Rebsamen et al., 2019; van Beek et al., 2019), physiotherapy exercise in others (Epelde et al., 2014; Im et al., 2015), cognitive-physical dual-task in Pedroli et al. (2018), neuropsychological testing in three studies (Fordell et al., 2011; Corno et al., 2014; Plechatá et al., 2019; Valladares-Rodriguez et al., 2019), gesture therapy in Morán et al. (2015) and Vanbellingen et al. (2017), and psychosocial support/educational in four studies (Tsai et al., 2012; Castilla et al., 2013; Cook and Winkler, 2016; Desteghe et al., 2017).

### Which Are the Theories and Tools Used to Assess VR?

A key component regarding the use and acceptance of technology is understanding elements that facilitate or reduce its use in terms of human factors, not only in terms of technical ones (Wildenbos et al., 2018). An architecture structure model, the user remote console (URC), was used as a theoretical background to design the physical training in Epelde et al. (2014), whereas the majority of the studies used psychological models to develop the VR systems. Technology acceptance model (TAM) and modified versions were used in most of the studies (Tsai et al., 2012; Wüest et al., 2014; Morán et al., 2015; Cook and Winkler, 2016; Money et al., 2019; Rebsamen et al., 2019; Valladares-Rodriguez et al., 2019), transformation of flow (ToF) in Pedroli et al. (2018), UX playability in two studies (Sáenz-de-Urturi et al., 2015; Valladares-Rodriguez et al., 2019), and user-centered design model (UCD) in Kiselev et al. (2015) and Brox et al. (2017). Importantly, some studies adopted technological theoretical frameworks adapted for older people, such as the senior UCD or the senior citizens' acceptance of information systems (SCAIS) (Brox et al., 2017; Nikitina et al., 2018). However, several studies did not report a theoretical model to design their systems (Kizony et al., 2006; Castilla et al., 2013; Corno et al., 2014; Schwenk et al., 2014; Im et al., 2015; Shubert et al., 2015; Desteghe et al., 2017; Trombetta et al., 2017; Vanbellingen et al., 2017; Plechatá et al., 2019; van Beek et al., 2019). Concerning the assessment of usability of VR systems, a wide range of quantitative and qualitative methods have been used (see Table 1 for specific information and Figures 5–7 for models and methods overviews). Concerning quantitative data, system usability scale (SUS) (Corno et al., 2014; Sáenz-de-Urturi et al., 2015; Shubert et al., 2015; Vanbellingen et al., 2017; Nikitina et al., 2018; Pedroli et al., 2018; Money et al., 2019; Rebsamen et al., 2019; van Beek et al., 2019), TAM-based questionnaires (Tsai et al., 2012; Wüest et al., 2014; Morán et al., 2015; Cook and Winkler, 2016; Rebsamen et al., 2019; Valladares-Rodriguez et al., 2019), UX questionnaires (Schwenk et al., 2014; Sáenz-de-Urturi et al., 2015; Brox et al., 2017; Desteghe et al., 2017; Rebsamen et al., 2019), UCD-based questionnaire (Brox et al., 2017), flow of experience scale (Pedroli et al., 2018), other usability questionnaires (Fordell et al., 2011; Epelde et al., 2014; Trombetta et al., 2017; Plechatá et al., 2019; Valladares-Rodriguez et al., 2019), adherence or motivation to training questionnaires (Im et al., 2015; Desteghe et al., 2017; Vanbellingen et al., 2017; Nikitina et al., 2018; Rebsamen et al., 2019; van Beek et al., 2019) or with VR data (Wüest et al., 2014; Kiselev et al., 2015; Desteghe et al., 2017; Vanbellingen et al., 2017; Nikitina et al., 2018; Rebsamen et al., 2019; van Beek et al., 2019), cybersickness assessment (Corno et al., 2014; Im et al., 2015; Plechatá et al., 2019), technology expertise (Corno et al., 2014; Rebsamen et al., 2019; Valladares-Rodriguez et al., 2019), and video analysis (Morán et al., 2015) were used. Regarding qualitative data, think aloud technique (Corno et al., 2014; Wüest et al., 2014; Shubert et al., 2015; Money et al., 2019; Rebsamen et al., 2019), heuristic evaluation or cognitive walkthrough (Castilla et al., 2013; Sáenz-de-Urturi et al., 2015), focus group (Castilla et al., 2013; Epelde et al., 2014; Kiselev et al., 2015; Brox et al., 2017; Desteghe et al., 2017), and semi-structured or structured usability post-experience interviews (Corno et al., 2014; Kiselev et al., 2015; Shubert et al., 2015; Brox et al., 2017; Vanbellingen et al., 2017; Pedroli et al., 2018; Money et al., 2019; van Beek et al., 2019) were used. The sense of presence was assessed only in three studies (Kizony et al., 2006; Nikitina et al., 2018; Pedroli et al., 2018).

Concerning the tools used, a variety of quantitative and qualitative methods are reported. However, it is important to remember that each of these instruments assess different aspects of usability and acceptance; some are more concerned about the task to perform (e.g., SUS) while others tap the emotional/motivational elements of the interaction (e.g., UX questionnaires) or the factors that hamper/facilitate the use of a technology (e.g., TAM-based tools). Qualitative tools are able to grasp different perspectives (individual or group) of the experience or the design by asking experts in the sector or the end-user itself. A multidimensional approach emerged in our search and should be preferred when selecting assessment tools.

### Are VR Clinical Systems for the Older People Usable?

In this section we outlined the findings of the included studies, reporting their strengths and weaknesses. Figure 8 shows mean and standard deviation for the available SUS scores, which display moderate to acceptable usability despite some cases of wide variation.

Cook and Winkler (2016) showed that OA find virtual environments (VE) from Second Life (SL) as feasible and applicable for healthcare purposes, especially for improving social interactions. Despite a high number of drop-outs, participants liked the realism and virtual experience (e.g., sports, changing avatar, teleporting, shopping) but bugs frustrated them and they found it hard to control the avatar and to learn SL. According to users, SL might be improved by clear training (i.e., individualized, small group), step-by-step teaching, by enlarging the screen, and facilitating the interaction. The exergame Falls Sensei was rated as engaging and usable for educating OA about risk fall (Money et al., 2019). Falls Sensei was rated as having a good usability (score SUS > 70, Bangor et al., 2009), especially by older users. Unified theory of acceptance and use of technology (UTAUT) thematic analysis on interviews (i.e., performance expectancy, effort, social influence) showed that users rated the training as a useful, positive experience, relevant for specific populations. Similarly, the Positive Bike (Pedroli et al., 2018) was rated as having good usability (mean SUS = 76.88, *SD* =17). Problems were found concerning the size of items on the screen and low realism or interaction users felt in the VE, but still had a positive experience and found the system useful. Stand Tall (ST) (Shubert et al., 2015) was rated by participants as having a nearly good usability (mean SUS = 65.5, *SD* = 21.2) and agreed in using ST to improve balance autonomously and accepted the Kinect sensor and the avatar. Senso system (Rebsamen et al., 2019) had high adherence, usability (mean SUS = 93.5, *SD* = 5.52), enjoyment, usefulness, and acceptability, also confirmed by think aloud technique. Similarly, van Beek et al. (2019) found optimal adherence and motivation toward their VR training. Despite some interaction issues with LMC and difficulty of the exercises, the system had marginal usability (mean SUS = 58.25, *SD* = 17.9) and was also rated positively at the interviews. Lineage was evaluated with high satisfaction by its users (Sáenz-de-Urturi et al., 2015). Gaming experience was positive, exercise adequate, and participants stated that they would use the game again. SUS improved across the three sessions (first mean SUS = 73.84, *SD* = 4.72; third mean SUS = 86.25, *SD* = 3.06). Acceptable usability was reported by OA and stroke patients for the TheraGame (mean SUS = 73.8, *SD* = 14.5) that also found the VR training adequate and enjoyable (Kizony et al., 2006). Good usability (first session mean SUS = 75.4, *SD* =13.8) was found by Vanbellingen et al. (2017) in their upper limb video game with a leap motion controller; however, usability did not change across the nine sessions. The training had a compliance of 87.4% and the adherence was rated as very good and remained stable across time. Users expressed that a 30 min session is the best time to not overload arm fatigue. Optimal (100%) adherence and good acceptance (e.g., ease, usefulness, intention to use) were found by Wüest et al. (2014). Nikitina et al. (2018) found that usability of the virtual gym App did not differ between groups with social interaction (mean SUS = 63, *SD* = 9) or interaction with coach only (mean SUS = 66, *SD* = 14). Moreover, the participants positively accepted the app, with high co-presence for the interaction group (interactions occurred especially with private messages), but adherence was similar for individual vs. group exercises with social support predicating adherence when social connections are low. Despite Corno et al. (2014) finding that virtual-multitasking test (V-MT) induces cybersickness symptoms, it was rated as usable (mean SUS = 69.17, *SD* = 8.2), the head-mounted display (HMD) was comfortable, interaction with the wand was difficult, instructions hard to remember, and realism sufficient. Similar results on HMD were found by Plechatá et al. (2019). HMD lead to the worst memory performances compared to non-immersive VR in OA, with users preferring neither desktop-based VR nor immersive VR, whereas young users liked immersive versions of the virtual supermarket shopping task (vSST). However, authors suggest non-immersive scenarios for OA. Fordell et al. assessed VR-DiSTRO, an immersive VR version of “paper and pencil” neglect neuropsychological battery, and showed that stroke patients tolerated and were engaged during the assessment, which was much faster than the classic evaluation (Fordell et al., 2011).

In order to design Game Up exergames and a senior-UCD model (Brox et al., 2017), it is crucial to involve older people and experts to create safe, fun, and usable games. Three-point Likert scale short questionnaires are suggested for end evaluations, whereas in the requirement, design, and implementation phases, interviews, observations, and group discussions are preferred for senior UCD. Similarly, in order to develop the Butler app (Castilla et al., 2013) it is important to gather information from end-users and experts from the first stage of the development and to create prototypes of the app. Graphics and navigations systems must be adequate and understandable for older people in order to reduce mental load. In the same way, the Health Buddies app (Desteghe et al., 2017) was initially designed with the end-users (AF patients and grandchildren). Participants, especially patients, were motivated to use the app but its usage decreased across 90 days. Despite adherence improving only in one patient, the UX with the app was easy to use and educational, and 60% of patients would use the app again. Experts and end-users of a joint rehabilitation virtual therapy were also involved in the evaluation phase in Epelde et al. (2014). Medical professionals and patients positively accepted the virtual therapist and training but patients stated that the avatar was too serious and lacked empathy. A team of experts developed an augmented reality exergame (Im et al., 2015), which did not have any side effects (e.g., cybersickness) and led to high adherence to the training.

The Interactive Trainer (Kiselev et al., 2015), despite some technical problems being reported, was evaluated according to interviews as easy to use, challenging, and motivating. Schwenk et al. (2014) assessed the gaming UX of a exergame with sensors, which was found to be effective, fun, easy to learn thanks to feedback, adequate, and well-designed. Interestingly, Valladares-Rodriguez et al. (2019) aimed at assessing UX and player eXperience (PX) of Panoramix neuropsychological touchscreen battery in OA, mild cognitive impairment (MCI), and Alzheimer's disease individuals. They found that Panoramix perception and acceptance were positive after the pilot study in the groups but was judged as more playable by OA, MCI, and AD in this order; nevertheless, PX improved after the second interaction in all groups. Additionally, administrators also evaluated the battery as playable, usable, useful, and with a good interface. Morán et al. (2015) used a TAM-based questionnaire and video analysis to assess usability. Users rated the VR gesture therapy (GT) as useful, easy, and with high UX and found that even technological expertise did not affect task performance. By analyzing verbal and non-verbal reactions, raters judged the system as more usable and fun for non-expert participants. Conversely, anxiety was low for expert users. Authors defined two approach strategies according to expertise, *explore-and-learn* and *score-and-complete*, respectively, for inexperienced and experienced participants that guided behaviors (e.g., anxiety, interaction strategies with the games) and reactions through the experience.

A comparison of semi vs. full immersive versions of Motion Rehab AVE 3D was done by Trombetta et al. (2017). Training was feasible for users and participants evaluated as important for usability feedback, third-person perspective, comfort (semi-immersion version), and immersion (full immersion). Authors suggested that, for post-stroke rehabilitation, semi-immersive systems are more comfortable than full-immersive VR. Tsai et al. (2012) showed that Sharetouch is a well-designed, easy, and usable system, independent of gender or age, and facilitates social interactions in OA. Importantly, significant effects of the rehabilitative training on different motor/physical measures were found in all the studies that tested efficacy and usability (Schwenk et al., 2014; Wüest et al., 2014; Im et al., 2015; Vanbellingen et al., 2017; Rebsamen et al., 2019; van Beek et al., 2019). However, risk of bias (see Supplementary Figure 1) is high for most of the categories (randomization, allocation, blinding, missing data, and reporting bias), as the majority of the research is quasi or non-experimental. Of note, the risk of incomplete data outcome was low.

In general, despite some technical weaknesses (e.g., realism, bugs), interaction constraints and physical/psychological barriers to technology use, the included VR studies showed that with adequate usability design methods, it is possible to develop effective and usable systems for clinical purposes in aging.

## Discussion

In the present paper we reviewed the current research on usability, user experience (UX), and feasibility of virtual reality (VR) clinical systems in older people.

Our work can be summarized in the following points: (1) most of the usability pilots involved healthy or heterogeneous diseased older people; (2) usability mainly concerned VR physiotherapy training; (3) most of the studies involved non-immersive scenarios; (4) quantitative (e.g., SUS) and qualitative (e.g., interviews) methods are the most used and suggested approach in usability piloting and technology acceptance model (TAM) is the main theoretical framework; (5) despite some interaction issues, VR systems are rated as having good usability by end-users.

Usability is a critical and complex task when specific end-users with particular needs are involved. Conditions that hamper the interaction with the device (Wildenbos et al., 2018), and also cultural and technology background, should be taken into account (Corno et al., 2014; Nikitina et al., 2018). For instance, Tuena et al. found that executive functions are overloaded by input device use in older people and this leads to worse memory performances (Tuena et al., 2019). Design guidelines should be used to avoid basic sensorimotor and interaction issues (see Phiriyapokanon, 2011; Loureiro and Rodrigues, 2014).

If, on the one hand, the studies included collected data from the target population (e.g., Parkinson's disease patients tested usability for Parkinson's disease rehabilitation), several others assessed usability with healthy older people or mixed-pathologies patients (e.g., Wüest et al., 2014; Sáenz-de-Urturi et al., 2015; Shubert et al., 2015; Trombetta et al., 2017; Vanbellingen et al., 2017); in this sense, diagnostic criteria were not clear or end-users characteristic do not match potential technology barriers of end-users. Future research should use strict inclusion/exclusion criteria according to diagnostic criteria of the diseases or syndromes. Moreover, in the context of healthcare, the end-users are also the medical professionals that use the technology with the patients. Usability should be assessed via questionnaires or interviews in the design and test phases (e.g., Castilla et al., 2013; Valladares-Rodriguez et al., 2019). Finally, despite some studies reporting the number of participants as a limitation (Corno et al., 2014; Desteghe et al., 2017; Vanbellingen et al., 2017; van Beek et al., 2019), a number of 5–10 individuals is sensible enough to identify a minimum of 80% circa of usability issues (Wüest et al., 2014; Brox et al., 2017).

The uses of VR systems in our review were mainly focused on motor rehabilitation. In healthcare, VR is mainly applied for the assessment and rehabilitation of sensorimotor, physical, and psychological deficits via non-immersive to immersive technologies (Lange et al., 2010; Bohil et al., 2011; García-Betances et al., 2015; Muratore et al., 2019; Tuena et al., 2019). We also encourage the use of pilot studies in other domains where VR is used for clinical purposes. For instance, it is important to evaluate usability of assessment tools (e.g., Pedroli et al., 2015; Desteghe et al., 2017). Mean usability session testing lasted 30 min; nevertheless, depending on the aims of the studies (e.g., memorability), longitudinal usability studies can be done as usability might improve after some sessions (Valladares-Rodriguez et al., 2019). Lastly, future research should focus more on immersive technology as technical development will lead to new forms of immersive VR and costs will be reduced. It is important to also assess these systems because they might lead to reduced cybersickness compared to desktop-based VR (Lange et al., 2010; Bohil et al., 2011; Plechatá et al., 2019).

Several studies (see Table 1) did not report a model on which usability and acceptance of a technology can be assumed. TAM-based and UX-based are useful for investigating and understanding psychological factors, whereas architecture design and user remote control (URC) are more useful for technical development. Indeed, usability, and in particular UX, are devoted not only to the ease of use and the technical bugs but also to the psychological domains (e.g., emotions, motivations; Vermeeren et al., 2010). However, as researchers in the context of aging face specific needs and barriers, adapted models with relevant variables should be used as the senior user-centered design (UCD) by Brox et al. (2017) or the senior citizens' acceptance of information systems (SCAIS) by Phang et al. (2006). Surprisingly, none of the authors used the senior technology acceptance model (STAM) by Chen and Shou (2014), which could be more suitable than TAM models not adapted to older people. Interestingly clinical researchers interested in technology usability, sense of presence, and clinical change may want to use the transformation of flow (ToF) theory, as presence and flow experiences might facilitate clinical change by means of VR (Riva et al., 2006).

Usability assessment (see Table 1 and Figure 7) tools should include a mix of quantitative methods (e.g., SUS, TAM-based questionnaires, UX-based questionnaires) and qualitative techniques (e.g., experience interviews, think aloud, heuristic evaluation). The systematic review on telemedicine systems by Klaassen et al. (2016) recommend SUS, TAM2, and PSSUQ and state that questionnaires along with interviews, which are both low-cost and flexible methods, can be used from early to final phases of usability. Indeed, questionnaires give useful quantitative data that, however, still need qualitative information to tap individual sources of variation. Therefore, a mixed approach composed of quantitative and qualitative tools is the preferred way to carry out complete, interpretable, and useful usability studies in older people. Additionally, we encourage a critical adoption of assessment tools according to the aims of the study, thus considering the aspects (e.g., individual, group, task, emotions/motivation, acceptance, adherence) to be engaged during the VR interaction.

Additionally, innovative quantitative techniques could be useful to track unexpected information about psychophysiological (e.g., eye-tracking, heart-rate, galvanic skin response, non-verbal communication) responses of the users to assess their affective and cognitive reactions to the VR system (Morán et al., 2015; Sáenz-de-Urturi et al., 2015). VR can also be used for evaluating usability and adherence (good >80%) by using time spent, number of log-ins, or interaction modality, giving additional quantitative data (Cipresso, 2015; Rebsamen et al., 2019; van Beek et al., 2019). Importantly, when testing immersive VR, cybersickness should always be assessed because it may negatively influence clinical practice and its reduction is a key objective of pilot studies (Kober et al., 2013; Corno et al., 2014; Tuena et al., 2017; Plechatá et al., 2019) and virtual embodiment with questionnaires if avatars are used (Kilteni et al., 2012; Gonzalez-Franco and Tabitha, 2018). Finally, in the early design phases, information from end-users (e.g., patients, medical professionals) could be gathered from group interviews or focus groups, where ideas from experts' opinions and needs can be used to guide VR development (Castilla et al., 2013; Brox et al., 2017). For instance, Brox et al. (2017) developed a senior-UCD with a mixed use of quantitative and qualitative methods to design a semi-immersive exergame for older people, through iteration from the early phases to the prototype. Researchers should be aware that step-by-step UCD (e.g., prototype development) and pretesting are critical for clinical VR settings (Novak, 2008; Im et al., 2015). However, we know that time is a limitation to some research projects and, in some occasions, there is no time for longitudinal and proper VR design. When this is not possible, we strongly encourage the use of qualitative and quantitative evaluation of the VR experience. In the same manner, it would be better to assess usability and acceptance separately from efficacy of a VR system, as quality of patients' healthcare services is intertwined with usability, acceptance, and adherence (Middleton et al., 2013).

Despite some technical and interaction issues (e.g., bugs, interaction difficulties, realism, sensors application), the included studies showed that usability of a wide range of VR clinical systems is good, well-accepted, adequate, effective, and useful. Skepticism of older people and digital divide are walls that could be successfully broken after the use of VR devices (Desteghe et al., 2017) and comfort of immersive VR can be improved by replacing visors with CAVE, although non-HMD systems are considered better for older people (Corno et al., 2014; Pedroli et al., 2018; Plechatá et al., 2019). Nevertheless, a recent study shows that OA positively accept and tolerate HMD VR (Huygelier et al., 2019). Indeed, Fordell et al. showed that stroke patients enjoyed the immersive VR assessment (Fordell et al., 2011). However, a future objective in the field is to make sensors application and use easier for this population, as home-based training, where no professional is present to provide assistance, is rising in popularity in VR clinical practice (Schwenk et al., 2014). Moreover, online assistance could be useful to help patients with set-up and exercises (Im et al., 2015; Nikitina et al., 2018). Morán et al. (2015) provided some guidelines concerning the feedback the VR training should give to older users:

- “*Provide timely feedback on successful actions in a simple and salient manner”;*
- “*Provide feedback on erroneous actions in a simple and salient manner*”;
- “*Provide simple and salient instructions on how to recover or solve an error*”;
- “*Provide feedback that fosters or inhibits specific behaviors from the user in a salient and concise manner*.”

Additionally, Teo et al. (2016) provide specific suggestions in their review for VR training in individuals with stroke-related impairments, such as flexible activity according to patients' objectives, possibility to adapt online the task by the therapist according to patient's needs, multiplayer services, and automated recording of patient tracking. Moreover, Teo et al. (2016) show that VR can be enriched with neurophysiological tools (e.g., EEG, fNIRS) that the researcher or the clinician can use to adapt the task according to individual effort or needs.

Finally, it is worth mentioning some solutions provided by the Cochrane guidelines (Higgins et al., 2011) that avoid risk of bias in usability experiments. Despite blinding procedures in cognitive/motor rehabilitation trials (VR vs. treatment as usual) being a hard task to fulfill, still randomization, attrition bias, and reporting bias can be improved, respectively, with random number generators, shuffling cards, or throwing dice, with adequate missing data manipulation (e.g., balanced observation, imputation) and via adequate hypotheses and primary/secondary outcomes specification in the introduction and then in the discussion and adequate analyses in the result section.

The present review outlined current VR usability piloting issues and strengths in healthy aging and age-related clinical conditions. In the following paragraph, we will provide suggestions for researchers who wish to run usability testing in the context of clinical application of VR systems for older patients.

## VR-usability Suggestions for the Older People (VR-USOP)

In the present paragraph we presented some suggestions we derived from findings of the systematic review. VR-USOP will be mainly focused on human-interaction factors rather than on technical aspects of developing VR clinical systems. Table 2 summarizes some suggestions in four steps to follow if researchers and clinicians wish to design and test their VR clinical apparatus to older end-users.

**Table 2:** VR-USOP.

1	Identify Barriers and Facilitators	•Use UTAUTM, STAM, MOLD-US or SCAIS models (Venkatesh et al., 2003; Phang et al., 2006; Chen and Shou, 2014; Wildenbos et al., 2018) •Clinical diagnosis and expert clinicians
2	Develop adequate VR and task	•Architecture design •Use older people technology design guidelines (Phiriyapokanon, 2011; Loureiro and Rodrigues, 2014) •Apply senior-UCD (Brox et al., 2017) •If training, use feedback guidelines (Morán et al., 2015) •Iterative prototyping
3	Define usability assessment	•Quantitative methods (e.g., SUS, TAM-based, UX-based questionnaires, PSSUQ) •Qualitative methods (e.g., post-experience interviews, think-aloud) •Additional methods (VR data, observation, psychophysiological measurements) •Assess usability and feasibility from medical professionals •If training, evaluate adherence •If immersive VR, evaluate cybersickness •If immersive VR with avatar, assess virtual embodiment •Consider PX for serious games and sense of presence for VR •Usability session should last 30 min approx.; more sessions can be included if there are experimental reasons
4	Test clinical use	•If usability results are unsatisfying, adjust VR system before clinical testing •If usability results are acceptable, start clinical efficacy testing

*PSSUQ, post-study system usability questionnaire; PX, player eXperience; SCAIS, senior citizens' acceptance of information systems; STAM, senior technology acceptance model; SUS, system usability scale; UCD, user-centered design; UTAUTM, unified theory of acceptance and use of technology model; UX, user eXperience; VR, virtual reality*.

The assessment of potential barriers and facilitators of the end-users, which can also include the medical professionals and technology acceptance models, is the first step. In our opinion, this is crucial as it allows the identification and the development of adequate characteristics of VR interaction and task (step 2). The latter aspects will be provided also by adopting architecture design, senior-UCD, and guidelines and prototyping, thus allowing the definition of usability assessment. In addition, we encourage ameliorating the methodology (risk of bias, see Supplementary Figure 1; i.e., randomization, allocation, blinding, manipulation of missing data, and reporting bias) to overcome the limitations of the available studies analyzed in the present review. VR usability and acceptance assessment should be defined and developed in accordance to the aims of the study (step 3). We suggest a mixed-approach with quantitative and qualitative methods (mainly focused on psychological experience of usability) and additional aspects to consider (see Table 2). Lastly, we suggest ensuring usability before clinical testing (step 4).

## Conclusions

This systematic review aimed at describing an overview of state of the art VR clinical systems for older people in relation to usability and providing researchers with suggestions based on the results of the review. Despite some limitations concerning the criteria used to recruit the samples, the low number of immersive technologies so far tested, and the high risk of bias of the studies, VR systems show good usability and acceptance among older people. A wide variety of quantitative and qualitative methods can be used to evaluate usability. We suggest adopting mixed-methodology with appropriate tools in order to grasp different aspects of the usability, acceptability, and user experience and to plan sessions according to objectives of usability. Piloting is a critical aspect of clinical studies with VR technology and we encourage future research to test usability of their applications following VR-USOP.

## Data Availability Statement

The datasets generated for this study are available on request to the corresponding author.

## Author Contributions

CT wrote the first draft of the manuscript. MS-B supervised and wrote the following drafts of the manuscript. MS-B, FG, CT, EP, AGal, and PT defined the methodology and objectives of the manuscript. MC assessed risk of bias and made the second search strategy. AGag gave framework for the VR-USOP. KG provided clinical expertise and support. GR and FL revised the manuscript. All authors contributed to the revision and final approval of the manuscript.

### Conflict of Interest

The authors declare that the research was conducted in the absence of any commercial or financial relationships that could be construed as a potential conflict of interest.

## Supplementary Material

Supplementary Figure 1 Risk of bias assessment.
