# Supplementary figures and images for: Usability Issues of Clinical and Research Applications of Virtual Reality in Older People: A Systematic Review
Source: Front Hum Neurosci. 2020 Apr 8;14:93. doi: 10.3389/fnhum.2020.00093 (PMC7156831; doi:10.3389/fnhum.2020.00093)

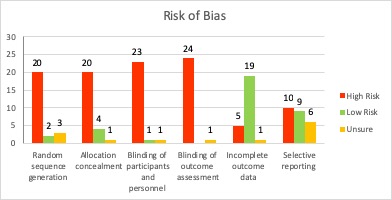

Supplement: Supplementary Figure 1 — Risk of bias assessment. [file Image_1.JPEG]
